# Pyroptosis, a double-edged sword during pathogen infection: a review

**DOI:** 10.1038/s41420-025-02579-6

**Published:** 2025-07-01

**Authors:** Yuanhang Zhang, Dengshuai Zhao, Tianyu Wang, Ping Li, Dixi Yu, Han Gao, Mengmeng Zhao, Limei Qin, Keshan Zhang

**Affiliations:** 1 Guangdong Provincial Key Laboratory of Animal Molecular Design and Precise Breeding, School of Animal Science and Technology, Foshan University, Foshan, 528225 China; 2 College of Veterinary Medicine, Nanjing Agricultural University, Nanjing, 210095 China

**Keywords:** Cell death, Microbiology

## Abstract

Pyroptosis, a distinctive form of programmed cell death (PCD) characterized by its inflammatory nature, is triggered by the activation of pore-forming proteins known as gasdermins (GSDMs). This process is marked by progressive expansion of a pore within the cell, ultimately leading to cellular membrane disruption and the substantial release of intracellular contents. Pyroptosis plays a pivotal role in the eradication of intracellular pathogen replication niches and in the modulation of the immune system through the release of danger signals. Emerging evidence suggests that viruses have developed sophisticated strategies to evade immune surveillance and establish persistent infections by manipulating host pyroptotic pathway This review presents recent advances on the mechanisms by which two major pathogens (virus and bacteria) activate or inhibit the pyroptosis process through their effector proteins, thereby facilitating their dissemination and blocking host immunity. These insights provide new perspectives on the regulatory mechanisms of interactions between hosts and pathogens in the pyroptosis process.

## Facts

- Pyroptosis exhibits a dual-edged sword characteristic: it eliminates intracellular pathogen replication niches and activates immunity by releasing danger signals, yet excessive inflammatory responses can also cause host tissue damage.
- Viruses and bacteria utilize their effector proteins to precisely activate or inhibit host pyroptotic pathways, thereby evading immune surveillance, promoting their dissemination, and establishing persistent infections.
- Evidence from in vivo studies demonstrates that drugs targeting cellular pyroptosis can effectively inhibit pathogen replication and attenuate inflammatory responses, highlighting their therapeutic potential.
- Newly discovered pyroptosis mechanisms (such as those mediated by NINJ1 and palmitoylated-GSDMD) suggest the existence of more unknown host–pathogen interactions.

## Introduction

Cell death is a prevalent physiological occurrence in animals that plays a crucial role in cell formation, maintaining cell homeostasis, immunity, and stress response. It includes both an accidental cell death, also known as ACD, and a purposeful cell death, also known as PCD. Cells’ ability to activate or inhibit PCD has far-reaching consequences for human life. Nonetheless, the system might overburden itself in situations where there is an abrupt and significant build-up of PCD, including during an infection, persistent inflammation, or tissue injury [1, 2]. Massive cellular contents are released into the extracellular space as a result of this uncontrolled PCD, thereby triggering the presence of danger-associated molecular patterns (DAMPs), which are signal molecules indicating damage. A robust immunological response is elicited when DAMPs are found in the extracellular space. This, in turn, attracts additional phagocytes and other immune cells to eliminate dangers and facilitate tissue healing. To manage an infection, the immune system triggers specific antimicrobial immune responses in response to pathogen-associated molecular patterns (PAMPs).

At present, the most extensively investigated forms of PCD are apoptosis, autophagy, necrosis, pyroptosis, and ferroptosis [3]. Among these, pyroptosis, an inflammatory form of PCD initiated by the identification of conserved PAMPs, is an important component of the host’s immune response to microbial infections and internal danger signals. Pore development, plasma membrane rupture, and pro-inflammatory cytokine activation and release define the cellular reaction to pyroptosis [4]. Gasdermin D (GSDMD), the executioner of pyroptosis, is cleaved into two fragments, the N-terminal and C-terminal portions, by caspases, in which the N-terminal domain infiltrates the lipid bilayer in the cytoplasm and assembles into pores in the cell membrane, resulting in osmotic imbalance. The N-terminal domain inserts into the lipid bilayer within the cytoplasm and oligomerizes to form pores in the cell membrane, which can induce osmotic imbalance, ultimately leading to cellular lysis and subsequent release of IL-18 and IL-1β [5–7]. Therefore, pyroptosis plays a crucial role in the resistance to pathogen attack [8, 9]. Although this simplified perspective conveys the sophisticated mechanics of pyroptosis during pathogen infection, current research has revealed new effectors and pyroptosis pathways, indicating a more complex and intertwined pattern of pyroptosis. This study focuses on recent breakthroughs in pyroptosis activation and the modification of immune responses to intracellular pathogens.

## Signaling pathways underlying pyroptosis

**Fig. 1:**
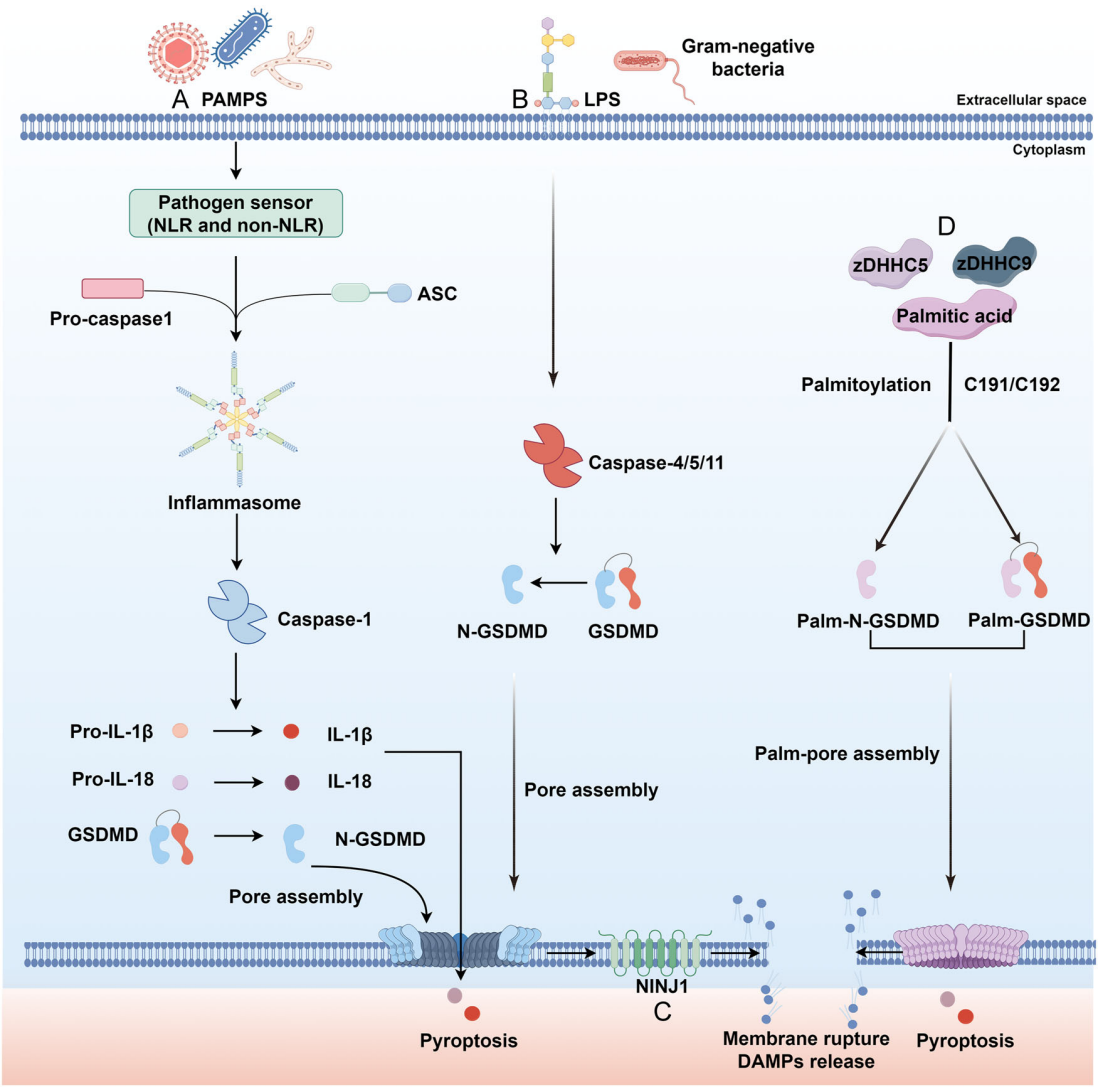
Signaling pathways of pyroptosis. **A** Classical pyroptosis pathway. **B** Non-classical pyroptosis pathway. **C** NINJ1-induced pyroptosis. **D** *S*-palmitoylated-GSDMD or its N-terminal induced pyroptosis.

Both the standard caspase-1-dependent pathway and the non-classical caspase-4, -5, and -11-dependent pathways are essential components of the pyroptosis signaling system (Fig. 1). In the classical caspase-1-dependent pyroptosis pathway, DAMPs such as bacteria or viruses initiate the typical pyroptosis cascade, which involves the inflammasome (AIM2), IFN-gamma inducible protein-16 (IFI16), and several NOD-like receptors. Cleavage of pro-caspase-1 follows from activation of inflammasomes, hence activating caspase-1. Activation of caspase-1 allows it to cleave GSDMD proteins, releasing the active N-terminal domain, which thereby encourages cell membrane perforation, which causes pyroptosis [5, 10, 11]. In the non-classical pathway, it has been found that signals such as bacterial lipopolysaccharide (LPS) can activate caspases 4 and 5 in humans and caspase-11 in mice. Subsequently, these caspases enzymatically cleave GSDMD, resulting in cellular pyroptosis [6, 12].

In addition to classical and non-classical pyroptosis pathways, recently, nerve injury-induced protein 1 (NINJ1), a cell surface protein with two transmembrane domains, has been discovered to play a role in the formation of GSDMD pore, which occurs after the disintegration of the plasma membrane during pyroptosis based on a forward-genetic screen technique [13]. Structural evidence indicates that NINJ1 produces filaments, thereby forming membrane pores in pyroptotic cells. The presence of these pores facilitates the release of large intracellular molecules, such as lactate dehydrogenase (LDH), as well as DAMPs like HMGB1 [14]. In terms of mechanics, the α3 and α4 TM helices of NINJ1 are initially found in the cell membrane when the cell is at rest. Then, a thermal transition trigger activates NINJ1, which probably allows the α1 and α2 helices to be inserted, resulting in the formation of a stable NINJ1 loop with a hydrophilic outer surface [15]. The ring acts like a cookie cutter, leaving many small holes in the cell membrane, causing membrane damage and shedding. Importantly, antibody-based NINJ1 oligomerization inhibition was found to decrease host inflammatory damage [16], showing a potential involvement of NINJ1 in pathogen infections.

Recent investigations have discovered that palmitoylation has a regulatory role in pyroptosis, adding to our understanding of a new regulatory route in innate immunity. These investigations have shown that the cleavage of GSDMD alone is not enough to produce the pores. Instead, GSDMD was S-palmitoylated at the C191 site upon inflammatory vesicle activation, and only the palmitoylated N-terminal region of GSDMD was capable of undergoing membrane translocation and hole formation [17–20], which suggested that palmitoylation was required for GSDMD activation. Hence, in the future, it is crucial to understand how pathogens manipulate GSDMD palmitoylation to control host cell pyroptosis and facilitate their infection. Additionally, the development of specific palmitoyl drugs holds significant potential for treating pathogen infections.

## Activation or inhibition of pyroptosis following viral infection

Once conserved PAMPs are identified by host PRRs, pyroptosis, a type of inflammatory cell death, takes place to control host antiviral immunity. Pyroptosis possesses a dual nature, as it serves as both a beneficial and detrimental process. On one side, its pro-inflammatory characteristics provide immune cells with an advantage in combating pathogens. This includes the recruitment of neutrophils to the site of infection. However, an overabundance of pyroptosis resulting from disrupted pathways can trigger the release of substantial quantities of inflammatory molecules, resulting in a cytokine storm that can induce severe clinical symptoms. This review presents a concise overview of the involvement of pyroptosis in pathogen infection, focusing on the mechanisms of activation and inhibition.

### Activation of pyroptosis during viral infection

**Fig. 2:**
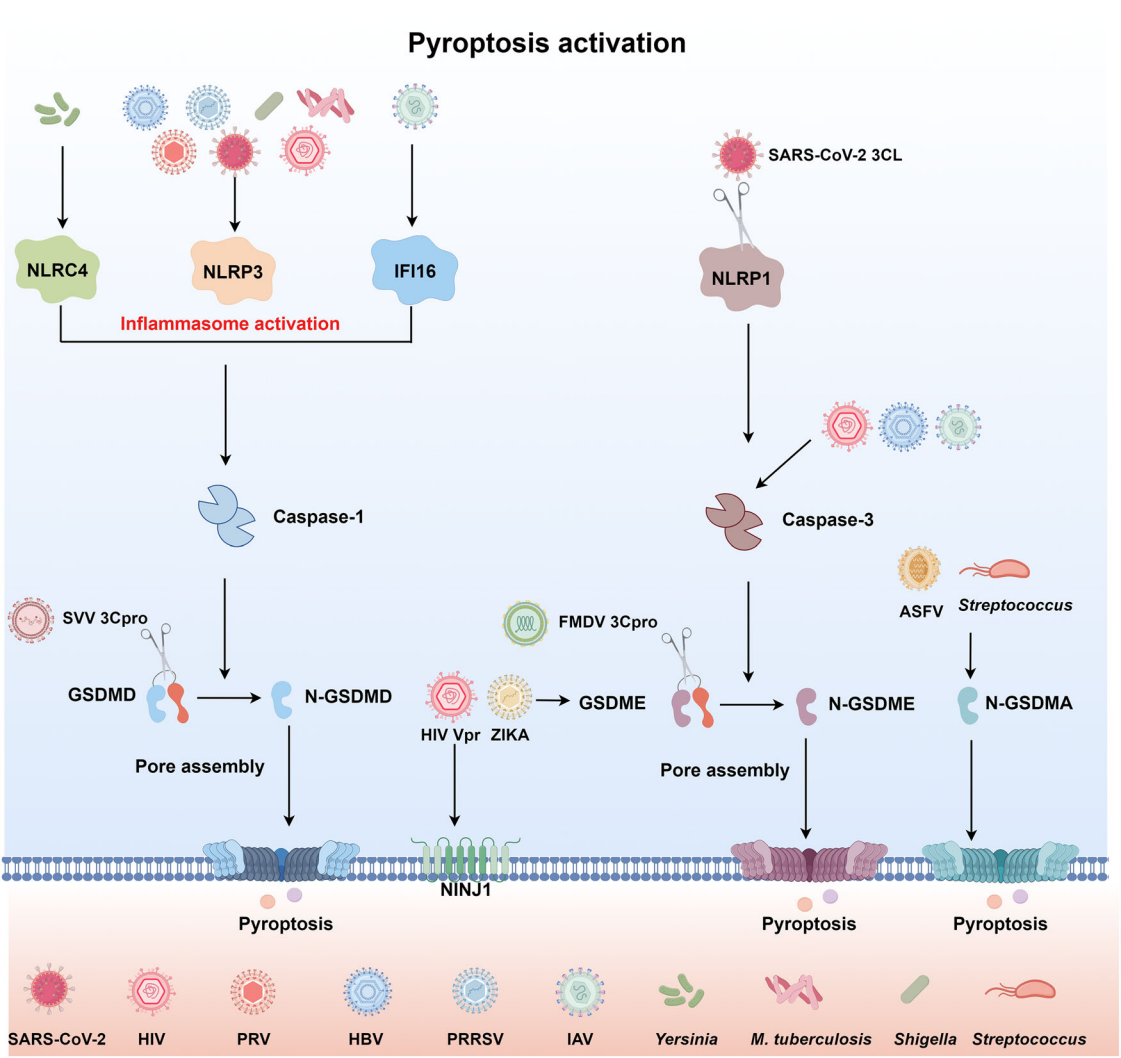
Mechanisms of viral activation of pyroptosis. Most viral/bacterial nucleic acids can stimulate pyroptosis through the activation of the NLRP1/3 inflammasome, NLRC3 inflammasome, and IFI16 inflammasome. Inflammasome activation stimulates the recruitment and activation of pro-caspase-1, resulting in the downstream cleavage of gasdermin proteins into C-terminal and N-terminal portions. The N-terminal gasdermin portion catalyzes pore formation and pro-inflammatory cytokine release. Several viral/bacterial proteins (including SVV/FMDV 3Cpro, SARS-CoV-2 NSP3, and Streptococcus SpeB) directly cleaved GSDMD/E/A into an N-terminal fragment to induce pyroptosis.

NLRP1, NLRP3, AIM2, and IFI16 inflammasome sensors regulate infection-induced caspase-1 activation and cleavage, allowing caspase-1-dependent pyroptosis [21]. In effector-triggered immunity, NLRP1 and NLRP3 recognized viral activity rather than viral molecules, while AIM2 and IFI16 induced pyroptosis upon identifying viral dsDNA (Fig. 2).

Coronavirus infections usually cause host hyperinflammatory disease in lungs and intestines, which is determined by a combination of viral infection and host inflammatory response [22, 23]. Consequently, inquiries into the potential connection between inflammatory vesicle activation, pyroptosis, and symptoms caused by the coronavirus have been initiated. Cellular pyroptosis is essential to the pathophysiology of COVID-19, as evidenced by the discovery of lysed GSDMD in lung tissue sections and BALF from COVID-19 patients, as well as the activation of the inflammasome by SARS-CoV-2 infection [24]. Subsequently, the specific mechanism of SARS-CoV-2-activated pyroptosis was reported. SARS-CoV-2 nucleocapsid protein (N) enhances the assembly and activation of the NLRP3 inflammasome via interacting with NLRP3, leading to cytokine storm and lung damage in mice, predicting the potential function of N proteins in SARS-CoV-2-induced pyroptosis [25]. Also, SARS-CoV-2 viroporins (ORF3a, E and M) activate NLRP3 inflammasome in human macrophage cell lines in macrophages to trigger pyroptosis-like cell death and IL-1α release from epithelial and endothelial cells [26]. In addition, the excessive production of SARS-CoV-2 NSP6 triggered the activation of caspase-1 via the NLRP3/ASC pathway, resulting in the maturation of interleukin-1β/18 and the occurrence of pyroptosis in lung epithelial cells. NSP6 engaged in direct interaction with the vacuolar ATPase proton pump component ATP6AP1, resulting in the inhibition of lysosomal-autophagic flow and then induced pyroptosis [27]. Particularly, the NLRP1 inflammasome, which is cleaved at the Q333 site by 3CL proteases and leads to inflammasome assembly, is crucial to the pyroptosis of SARS-CoV-2-infected human lung epithelial cells. Thereby, the caspase-3/GSDME pathway was utilized to enhance pyroptosis [28]. Recently, Grin et al. discovered that 3CLpro and nucleocapsid protein were released by unconventional secretion from SARS-CoV-2-infected cells through caspase-activated GSDMD and GSDME pores. The 3CLpro inactivated GSDMD to prevent excessive pore formation and pyroptosis, which further enriched the vital role of 3CLpro in pyroptosis caused by SARS-CoV-2 [29]. The mouse hepatitis virus (MHV) also triggers the NLRP3 inflammasome, leading to inflammatory cell death known as panoptosis, and the absence of GSDMD promotes the activation of the NLRP3 inflammasome and initially reduces cell death [30]. Porcine enterocoronaviruses, including TGEV and PDCoV, mainly infect the small intestinal epithelium of neonatal pigs with high morbidity and mortality. TGEV infection of the intestinal epithelium resulted in the production of pro-IL-1β and cleavage of GSDMD, which induced pyroptosis, and the absence of GSDMD inhibited the replication of TGEV and PDCoV in vitro [31]. In general, coronaviruses have developed a variety of mechanisms to induce cellular pyroptosis, thereby facilitating their persistent infection in vivo, which is also commonly associated with the severe inflammation caused by coronaviruses. This indicates that a combination of NLRP3 inhibitors and protease inhibitors may prove an efficacious treatment for patients with COVID-19.

Although combination antiretroviral therapy (cART) has been successful, it is important to note that human immunodeficiency virus-1 (HIV-1) infections continue to negatively affect immunological function and cause neuronal death, resulting in the development of HIV-associated neurocognitive disorders (HAND). HAND is now well acknowledged as an intricate neurodegenerative disorder marked by extensive, advancing, hastened neuronal demise and cognitive decline [32]. A preliminary study on HIV-1 found that caspase-3-mediated apoptosis was responsible for just a small portion of CD4 + T cell mortality resulting from HIV-1 infection. The majority, approximately 95% or more, of CD4 + T cells underwent caspase-1-mediated pyroptosis [33]. Another study conducted in the same year demonstrated that IFI16 acted as a host DNA sensor essential for inducing pyroptosis in CD4 + T cells due to incomplete HIV infection. Later, it was found that the HIV-encoded gp120 protein induced microglial NLRP3-dependent pyroptosis and IL-1β synthesis [34]. However, the activation of microglial NLRP3 inflammasome was found to reduce the production of neuroinflammatory factors and neuronal damage caused by gp120. This suggests that targeting NLRP3 could be a potential therapeutic approach for treating HIV-associated HAND [35]. HIV-encoded viral protein R (Vpr) produced time-dependent cleavage of GSDME and NINJ1 activation with concomitant cell lysis, revealing a new mechanism by which HIV infection promoted pyroptotic death in neurons [36]. In addition, the frontal cortex tissues from HIV-infected macaques were analysed, which exhibited increased levels of GSDME and NINJ1 expression in cortical neurons. Significantly, its expression was found in the same location as caspase-3 detection in rats suffering from neurological illness [36]. Therefore, pyroptosis has been identified as a crucial factor in the advancement of HAND, intensifying the harm to neurons and cognitive impairments seen in affected individuals.

Humans are highly infected with herpes simplex virus type 1 (HSV-1), which can lead to severe inflammations such as herpes simplex encephalitis (HSE). HSV-1 causes GSDMD-dependent pyroptosis via activating the NLRP3 inflammasome in murine microglia, resulting in mature IL-1β and active caspase-1. Inhibiting microglial NLRP3 inflammasome activity reduces this effect [37]. Furthermore, there have been reports indicating that the infection of human neuron-like SH-SY5Y cells and primary human and murine brain cells with lytic HSV-2 results in pyroptosis mediated by GSDME, which is triggered by the activation of an ER stress-driven pathway [38]. Pseudorabies virus (PRV), which belongs to the herpesvirus genus, has been found to activate the NLRP3 and IFI16 inflammasomes. This activation leads to pyroptosis, specifically mediated by GSDMD and not GSDME [39]. A further study has demonstrated that the PRV nonstructural protein UL4 is responsible for the promotion of pyroptosis through the enhancement of ASC-dependent inflammasome activation [40]. Conversely, a deficiency of the viral UL4 has been shown to result in a reduction in ASC-dependent inflammasome activation and pyroptosis. Overall, these findings indicate that both GSDMD-mediated pyroptosis and GSDME-mediated pyroptosis are significant factors in HSV1/2 infection and offer potential therapy targets for viral inflammation.

Hepatitis B virus (HBV), belonging to the viral hepatitis family, has the potential to cause chronic illness. HBV frequently induces renal inflammation, in addition to hepatitis. In response to oxidative stress, HBV X protein activates the NLRP3 inflammasome, leading to pyroptosis and the release of IL-1β, IL-18, and other components [41]. Another study recently demonstrated that activation of DR5 by HBV e antigen (HBeAg) led to death of M1-like and M2-like macrophages, as well as pyroptosis of the former, revealing a unique action of HBeAg [42]. Additionally, the lack of MHC-I in HBV-reactivated hepatocytes triggered cytotoxic NK cells, which subsequently worked in a manner reliant on granzyme and perforin to cause hepatocyte pyroptosis via the GSDMD/caspase-8 axis [43]. Furthermore, it has been documented that the duck hepatitis A virus type 1 (DHAV-1) infection triggers pyroptosis in duck embryo fibroblasts by initiating caspase-3 cleavage of GSDME [44]. More precisely, the process of cell death known as pyroptosis, produced by DHAV-1, was controlled by the contact between the first 130 amino acids of the 2A2 protein and the structural domain of MAVS called TM [44].

Pandemic and seasonal influenza A virus (IAV) pose a significant continuous hazard to human health worldwide. Severe IAV infections, characterized by high inflammation, cell death, and epithelial destruction, contribute to the development of untreatable and deadly lung illness [45, 46]. The initial findings indicated that the detection of IAV proteins NP and PB1 by ZBP1 could activate pyroptosis and inflammatory reactions through the RIPK1-RIPK3-Caspase-8 pathway [47]. Another study observed that the influenza virus caused programmed cell death, known as apoptosis, in the early stage of infection. However, in the later stage, the virus triggered pyroptosis in normal or precancerous human bronchial epithelial cells, which was controlled by the signaling of type I interferon [48]. Not only was IFI16 found to be an IAV RNA sensor by interacting with genomic RNAs, but activation of IFI16 produced type I and type III IFN signaling and pro-inflammatory cytokines *via* the STING-TBK1 and pro-caspase-1 signaling axes, so promoting pyroptosis [49]. It was now abundantly evident that pyroptosis triggered by the influenza virus was caused in great part by the interferon signaling system. Also, the GSDMD deficiency has been demonstrated to markedly diminish neutrophil infiltration of the airways, along with the levels of pro-inflammatory cytokines, and enhance influenza resistance, reduce the viral burden, and mitigate the severity of lung lesions, including a reduction in epithelial damage and cell death [50]. Additionally, it was discovered that the H7N9 virus replicates effectively in the lungs of mice, causing the activation of GSDME-mediated pyroptosis in alveolar epithelial cells. This leads to the release of cytosolic contents, which in turn triggers a cytokine storm [51]. These investigations have shown that an excessive inflammatory response and damage to the tissues after severe infection with IAV might lead to a fatal lung disease.

Recently, there have been additional reports of virus-induced pyroptosis. For example, the Zika virus (ZIKV) activated the executor GSDME in vitro and in vivo, causing placental cells to undergo pyroptosis, which played a crucial role in the negative effects on fetal development produced by ZIKV [52]. A study has demonstrated that host transcriptional repression of Rift Valley fever virus (RVFV) nonstructural protein NSs resulted in the rapid downregulation of myeloid cell leukemia-1 (MCL-1), which in turn led to BAK activation in mitochondria. This process then triggers the production of mtROS and the subsequent release of oxidized mitochondrial deoxyribonucleic acid (ox-mtDNA) into the cytoplasm, and activates the NLRP3 inflammasome, which in turn triggers NLRP3-GSDMD-mediated pyroptosis infected with RVFV [53]. Lin et al. provided evidence that Oncolytic parapoxvirus ovis (ORFV) and its modified therapeutic versions can induce tumor pyroptosis through GSDME. This study emphasizes the important role of GSDME-mediated pyroptosis in the immune response against tumors using oncolytic ORFV and identifies potential combined therapeutic approaches for cancer treatment [54]. NLRP3 inflammatory vesicles have been reported to be activated by the severe fever with thrombocytopenia syndrome virus (SFTSV) as a result of the interaction between the viral nonstructural protein NSs and its N-terminal fragment. As a result, pyroptosis and the release of IL-1β are induced. [55]. Also, it was proved that infection with porcine reproductive and respiratory syndrome virus (PRRSV) induced pyroptosis and the secretion of IL-1β by activating the NLRP3 inflammasome. Mechanistically, PRRSV infection caused the trans-Golgi network (TGN) to disintegrate, which served as a platform for the activation of NLRP3 [56]. African swine fever virus (ASFV) infection is known to cause pyroptosis, and it can be managed by cleaving the pyroptosis execution protein GSDMA. This is a novel molecular mechanism for controlling pyroptosis. [57]. The nonstructural protein 1 (NS1) of the dengue virus (DENV) can activate inflammasomes and release IL-1β in a manner that depends on caspase-1. This mechanism occurs independently of inflammasome pathways, but it does require the presence of CD14 for activation [58]. Fascinatingly, DENV NS1-induced inflammasome activation neither caused pyroptosis nor fast cell death, suggesting that DENV might induce pyroptosis in an inflammasome-independent manner [58]. Coxsackievirus A16 (CV-A16) and A10 (CV-A10) infections promote pyroptosis mediated by NLRP3 and increase the release of inflammatory cytokines. The activation of NLRP3 speeds up the development of pyroptosis and worsens the inflammatory response, indicating that NLRP3-driven pyroptosis may play a role in the synthesis of CV-A16 and CV-A10 in SH-SY5Y cells [59].

In addition to inflammasome-mediated pyroptosis, viruses have also been observed to induce pyroptosis by directly cleaving GSDMD/E into an N-terminal fragment with their own encoded proteases. For instance, Seneca Valley Virus (SVV) infection caused pyroptosis in SK6 cells by both caspase-dependent and caspase-independent mechanisms. The protease activity of 3Cpro specifically targeted porcine GSDMD (pGSDMD) for cleavage, resulting in the generation of pGSDMD1-277, which then triggered pyroptosis [60]. Subsequent research revealed that FMDV 3Cpro enzyme also splits porcine GSDME (pGSDME) at the Q271-G272 junction, which is close to the cleavage site (D268-A269) of pCaspase-3. This process triggers pyroptosis [61]. Recent reports also indicated that the SARS-CoV-2 protease NSP3 triggered the activation of GSDME-mediated pyroptosis by cleaving GSDME at residue G370 [62]. The single-protein signaling pathways are rare, particularly in contrast to the activation of GSDMs that necessitates the presence of caspases and inflammasome sensor proteins. The one-protein recognition-effector system may have evolved to be more effective at triggering host defense responses, inflammation, and cell death. However, a limitation of this system that relies on a single protein is that infections have the potential to utilize their own virulence factors to impede the function of the host protein, so shifting the balance of host–pathogen interactions in favor of the pathogen. Furthermore, this singular protein pathway possesses a restricted capacity to detect a diverse range of infections and indications of danger.

### Inhibition of pyroptosis during viral infection

**Fig. 3:**
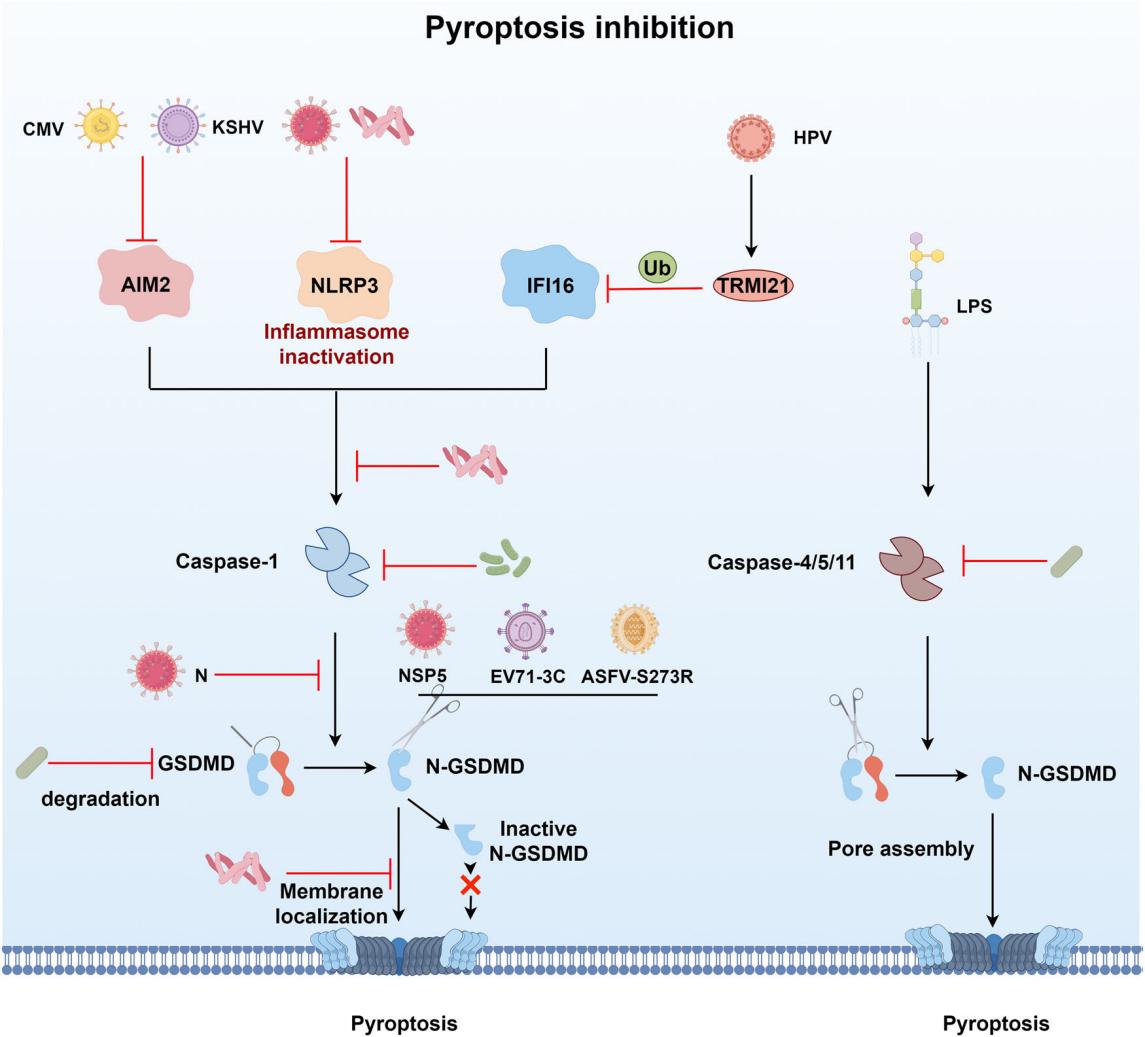
Mechanisms of viral antagonism of pyroptosis. Viral/bacterial proteins (SARS-CoV-2 N/PLP, KSHV SOX, CMV M84, *Mycobacterium tuberculosis* PKnF, *Salmonella* virulence C) prevent inflammasome assembly to inhibit pyroptosis. Also, several viral/bacterial proteins (SARS-CoV-2 N/NSP5, PEDV NSP5, ASFV S273R, EV71 3C, *M. tuberculosis* PtpB, *Shigella* IpaH7.8) directly interact with GSDMs to inhibit pyroptosis.

Overall, virus-induced pyroptosis is often associated with clinical diseases associated with severe inflammation in their hosts, yet pyroptosis inhibits viral replication in vivo to some extent due to its pro-inflammatory nature. Therefore, some viruses have also evolved strategies to inhibit pyroptosis to promote viral replication in vivo (Fig. 3). In contrast to one study showing that SARS-CoV-2 nucleocapsid proteins can activate NLRP3 inflammatory vesicles and promote pyroptosis [25], it was interesting to note that almost simultaneously, another study reported that SARS-CoV-2 nucleocapsid binds to the GSDMD junction region and prevents GSDMD processing by caspase-1, thereby inhibiting GSDMD cleavage and host cell pyroptosis in vivo and vivo [63]. Nevertheless, both investigations depended on SARS-CoV-2 N protein overexpression systems. It is crucial to confirm these findings by utilizing in vivo infection models in order to resolve these contradictory outcomes. Later, it was noticed that the SARS-CoV-2 papain-like protease (PLP) had a negative effect on the NLRP3 inflammasome pathway. It suppressed the release of IL-1β and reduced the caspase-1-mediated pyroptosis of human monocytes [64]. The human papillomavirus (HPV) utilizes the E3 ligase TRIM21 to facilitate the process of ubiquitination and degradation of the IFI16 inflammasome. This action effectively hinders pyroptosis by activating inflammatory vesicles and promoting the production of IL-18 and IL-1β to free itself from immune surveillance [65]. Kaposi’s sarcoma-associated herpesvirus (KSHV) ORF37-encoded SOX protein suppressed AIM2 inflammasome activation by interacting with the AIM2 HIN domain and disrupting AIM2: dsDNA polymerization and ASC recruitment and oligomerization, which promoted KSHV lytic replication and inhibited pyroptosis [66]. Cytomegaloviruses (CMVs) have been shown that the M84 protein inhibit the release of pro-inflammatory cytokines and pyroptosis. Mechanically, M84 inhibits inflammasome formation by interacting with the pyrin domain of AIM2 and ASC, hence blocking caspase-1-mediated activation of IL-1β, IL-18, and GSDMD [67].

In addition to targeting inflammasomes to inhibit pyroptosis, viruses have similarly evolved strategies to inhibit pyroptosis by directly targeting GSDMDs. For instance, a study showed that the NSP5 protein of coronaviruses (PEDV, PDCoV, SARS-CoV-2, and MERS-CoV) possessed protease activity that cleaved human and porcine GSDMD into two fragments incapable of inducing pyroptosis, providing strong evidence that may utilize the NSP5 protein to inhibit host cell pyroptosis, thereby facilitating their replication within the host organism [62, 68]. ASFV and Enterovirus 71 (EV71) have demonstrated a comparable mechanism via the viral proteases S273R and 3C, respectively [69, 70]. Different from its role in GSDMD-mediated pyroptosis activation, another study reported that picornavirus SVV protease 3C cleaved both pGSDMA and hGSDMA, generating a shorter fragment that fails to associate with the plasma membrane and does not induce pyroptosis. This cleavage by SVV 3C suppresses GSDMA-mediated lactate dehydrogenase release, bactericidal activity, and lytic cell death [71]. Generally, the promotion of viral replication in vivo is dependent on viral suppression of the GSDMD- and inflammasome-pyroptosis pathway, which helps identify potential targets for antiviral medication development.

## Activation or suppression of pyroptosis following bacterial infection

### Activation of pyroptosis during bacterial infection

Bacterial pathogens, like viral infections, can alter the host’s pyroptosis pathway to achieve immune escape and facilitate infection. Several pathogenic bacteria release effector proteins that stimulate inflammatory vesicles via different mechanisms, establishing an inflammatory response and initiating the septic process (Fig. 2). Viruses predominantly subvert it through virulence factors targeting gasdermins or inflammasomes, while bacteria often activate pyroptosis via PAMPs. This section will specifically address bacterial pathogens, such as *Mycobacterium tuberculosis*, *Shigella*, *Yersinia*, and *Salmonella*, which frequently induce hyperinflammation in the host. The bacterial pathogens mentioned possess different secretion systems, such as the type III secretion system (T3SS) present in *Shigella*, *Yersinia*, and *Salmonella* and the type VII secretion system (T7SS) found in *Mycobacterium* TB, which act as PAMPs to trigger the inflammasome-pyroptosis pathway or transport various effector proteins to target host regulators involved in the process of pyroptotic cell death. A more comprehensive explanation of these diverse pathogenic techniques for regulating pyroptosis enhances our comprehension of the pathogenesis of bacterial infections.

*Mycobacterium tuberculosis* is the pathogenic microorganism responsible for causing tuberculosis, a significant and ongoing worldwide public health concern [72]. In contrast to other bacterial diseases that employ toxins to directly destroy host cells, the intracellular pathogen *M. tuberculosis* utilizes a multitude of effector proteins to regulate host cell death, including pyroptosis [73, 74]. The preliminary results revealed that ESX-1, the T7SS of *Mycobacterium* TB, caused harm to phagosomes, resulting in the release of molecular patterns from phagosomes carrying *M. tuberculosis*. Consequently, the pathways of inflammasome-pyroptosis are activated [75]. The ESX-1-dependent degradation of the plasma membrane triggers the activation of the NLRP3 inflammasome by the release of potassium ions, resulting in pyroptosis [76]. Afterwards, it was found that some mycobacterial proteins, such as PE_PGRS19, EST12, and PPE13, activate the host inflammasome-pyroptosis pathways [77–79]. The findings of these studies collectively indicate that the inflammasome-focal pathway plays a pivotal role in the hyperinflammation associated with *M. tuberculosis* infection. This observation offers a compelling rationale for the potential therapeutic utility of drugs that target the inflammasome in the management of *M. tuberculosis* infection.

*Shigella*, a gram-negative enteric bacterium, causes acute inflammation and mucosal damage in humans and primates, leading to severe gastrointestinal disorders [80]. Rapid phagocytic vesicle membrane breakdown, resulting in macrophage pyroptosis and bacterial multiplication, is caused by the *Shigella* T3SS. For instance, the T3SS components MxiH and MxiI trigger the NLRC4 inflammasome, resulting in macrophage pyroptosis upon recognition by the macrophage’s cytoplasmic immunological sensors, the NAIPs [81, 82]. *Shigella* IpaH7.8 degrades host glomulin, which triggers the activation of the NLRP3 and NLRC4 inflammasomes and leads to macrophage pyroptosis [83, 84]. Furthermore, it has been demonstrated that IpaH7.8 ubiquitinates and degrades murine NLRP1B, releasing the C-terminal region that is active and aids in the production of inflammasomes and macrophage pyroptosis.

*Yersinia* species are a type of Gram-negative enterobacteria that have the ability to infect humans and cause a diverse range of diseases, ranging from mild gastroenteritis to severe and potentially fatal plague [84]. There is evidence that the *Yersinia* T3SS can trigger pyroptosis in hosts through the inflammasome [85]. The mechanism for the activation of the NLRC4 inflammasome and subsequent pyroptosis involves the recognition of the T3SS in Gram-negative bacteria, specifically *Yersinia*, by cytoplasmic immunological sensors called NAIPs [86, 87]. Furthermore, the *Yersinia* T3SS effectors YopE and YopT interfere with the activation of Rho GTPase in host cells, leading to the induction of pyroptosis through the Pyrin inflammasome [88]. The *Yersinia* translocators YopB and YopD, which are integral membrane proteins that are inserted into the plasma membranes of host cells, activate the NLRP3 inflammasome upon translocation into the host cells [89]. YopB and YopD cause instability in the vacuoles of macrophages hosting *Yersinia* bacteria. This leads to the recruitment of galectin-3 and GBPs to the bacteria, potentially assisting in the activation of caspase-4 noncanonical inflammasome and the process of pyroptosis triggered by LPS [90].

*Salmonella*, a Gram-negative, facultative intracellular bacterium, can cause gastroenteritis to typhoid fever depending on the host and serotype [91]. *Salmonella* infections can result in pyroptosis mediated by the host NAIP/NLRC4-inflammasome through their flagellins and T3SS components, which include the needle complex proteins PrgI and PrgJ [92]. Alternatively, *Salmonella* LPS activates caspases 4 and 5 (human) as well as caspase-11 (mouse), which then leads to cleavage of GSDMD, subsequently resulting in pyroptosis [12]. Futhermore, group 3 innate lymphoid cells (ILC3s) infected with enteric S. Typhimurium undergo caspase-1/GSDMD-mediated pyroptosis, which reduces the number of ILCs and produces IL-22, a cytokine that S. Typhimurium uses to induce host dysbiosis and ultimately limit bacterial colonization [93].

*Streptococcus pyogenes*, commonly referred to as group A Streptococcus (GAS), is a prevalent skin infection that leads to substantial illness and death worldwide [94]. Streptococcal infections trigger the activation of the NLRP3 inflammasome, leading to pyroptosis. Both of these processes have been linked to host inflammation and autoimmune responses. One example is that *S. pneumoniae* induces cellular damage by the production of reactive oxygen species (ROS), which triggers the activation of the NLRP3 inflammasome and results in caspase-1-dependent pyroptosis. And this process is hindered by autophagy [95]. M protein is the most prevalent GAS surface protein, and GAS strains with the M1 serotype have been related to invasive illnesses. The release of soluble M1 protein in macrophages can activate caspase-1-dependent NLRP3 inflammasomes, which in turn lead to the release of the pro-inflammatory cytokine IL-1β and the pyroptotic death of macrophages [96]. Two investigations virtually simultaneously found that the GAS cysteine protease SpeB virulence factor cleaves GSDMA and generates a strong N-terminal fragment, implying that GSDMA can function as a guard protein directly detecting pathogenicity [97, 98]. These findings imply that GSDMA, present in different skin cells, serves as a molecular barrier preventing dangerous germs from causing severe skin diseases.

### Inhibition of pyroptosis during bacterial infection

*Mycobacterium tuberculosis* has developed mechanisms to hinder the inflammatory vesicle-focused route, therefore enabling evasion of the immune system (Fig. 3). For example, *M. tuberculosis* Zmp1, a presumed Zn^2+^ metalloprotease, has been demonstrated to hinder caspase-1 activation and IL-1β generation, therefore enhancing bacterial viability in mice [99]. Moreover, PknF, a different protein from Mycobacterium, has been found to be a eukaryotic-like serine/threonine kinase that inhibits the activation of the NLRP3 inflammasome and pyroptosis [100]. Specifically, the protein PtpB, which is secreted by *M. tuberculosis*, binds to host ubiquitin in order to enhance its phosphoinositide phosphatase activity. This activity enables PtpB to target and dephosphorylate host plasma membrane phosphoinositides, thereby preventing pyroptosis and enabling *M. tuberculosis* immune evasion by so limiting the membrane localization of GSDMD-N [101].

Despite the ease with which classical inflammasomes can activate macrophages for pyroptosis, bacteria can also impede epithelial cell death by eliminating non-classical inflammasomes to establish an intracellular replication niche. *S. flexneri* effector OspC3 inhibits caspase-4 p20 subunit heterodimerization with p10 subunit, preventing caspase-4-dependent pyroptosis and promoting bacterial epithelial invasion [102, 103]. Further research has shown that OspC3 promotes arginine ADP-riboxanation, a unique post-translational modification of caspase-4/11, which prevents the autoprocessing of caspase-4 instead of impeding its heterodimerization to impede GSDMD cleavage [104].

*Shigella* manipulates host pyroptosis by introducing a set of T3SS effectors that possess E3 ubiquitin ligase activity. These effectors, referred to as IpaH family proteins, are active in epithelial cells. In contrast to its involvement in the initiation of pyroptosis, the *Shigella* effector IpaH7.8 has been demonstrated to ubiquitinate human GSDMD to promote degradation, rather than mouse GSDMD, in order to inhibit pyroptosis [105]. Furthermore, recent studies have shown that *Shigella* IpaH9.8 is capable of ubiquitinating and degrading GBPs [105], working together with OspC3 to restrict pyroptosis in IFN-γ-primed epithelial cells [106]. These findings highlight how *Shigella* has evolved to specifically adapt to different species, allowing it to infect humans by interfering with pyroptosis in epithelial cells.

*Yersinia*’*s* pathogenicity is also linked to Yops’ suppression of host pyroptosis. *Yersinia* YopK may prevent YopB and YopD from entering host cells, preventing both canonical and noncanonical inflammasome-mediated pyroptosis [89]. YopM, a crucial factor for *Yersinia’s*ability to cause disease, has been shown to directly attach to caspase-1 and prevent its activation, which effectively hinders caspase-1-dependent pyroptosis and facilitates bacterial infection in vivo [107].

New data suggest that *Salmonella* uses tactics to avoid the immune response mediated by pyroptosis. For example, *Salmonella virulence* C (SpvC), a crucial component of *Salmonella virulence* that is highly similar to S. flexneri OspF, was discovered to inhibit the autophagic flux in a way that was reliant on its phosphothreonine lyase activity. This, in turn, prevented NLRC4/NLRP3-driven pyroptosis [108, 109]. A further study has revealed that the human-adapted *S. paratyphi* strain expresses FepE, a polysaccharide copolymerase, which is responsible for the synthesis of exceptionally long O-antigen chains. These chains have the capacity to inhibit macrophage pyroptosis, which is likely due to the steric hindrance they present to caspase 4-mediated recognition of bacterial lipid A [110]. Interestingly, *S. paratyphi* has a significantly greater expression level of FepE during infection than S. Typhimurium, and as a result, causes substantially less pyroptotic cell death [110].

## The therapeutic implications of activators or inhibitors of pyroptosis

GSDMD exhibits broad expression across both neoplastic and non-neoplastic pathologies, with emerging evidence highlighting its therapeutic potential through targeted inhibition. Current pharmacological strategies predominantly focus on the evolutionarily conserved cysteine residue (Cys191 in humans/Cys192 in mice) to disrupt two critical processes: GSDMD proteolytic activation and N-terminal oligomerization. This dual mechanism effectively blocks pyroptotic pore formation and subsequent inflammatory cascades.

Notable small-molecule agents, including necrosulfonamide, disulfiram, and fumarate derivatives, demonstrate conserved binding specificity for this cysteine motif. Their therapeutic efficacy manifests through distinct pathophysiological models-from reducing sepsis mortality to ameliorating neuroinflammation in EAE/MS paradigms [111–113]. Interestingly, the pharmacological landscape reveals functional dichotomy: while most compounds (e.g., DMF, NU6300) suppress GSDMD activation via cysteine modification to mitigate conditions like colitis and autoimmune encephalitis [114], the selective agonist DMB paradoxically enhances pore assembly at Cys191 to potentiate anti-tumor immunity through controlled pyroptosis induction [115]. Beyond direct binding to the Cys191/192 site, other inhibitors such as LDC7559 and tea polyphenol nanoparticles (TPNs) exert dual suppression on both GSDMD-N activation and supramolecular assembly. These interventions demonstrate multi-organ protective effects in sepsis models, including enhanced survival metrics, thermoregulatory stabilization, and functional preservation across vital organ systems [116, 117].

Notwithstanding promising preclinical outcomes spanning inflammatory bowel disease, neuroinflammatory disorders, and oncology models, translational progress remains constrained. No GSDMD-targeting agents have advanced to clinical trials or received regulatory approval, underscoring critical gaps in pharmacodynamic optimization and safety profiling that warrant systematic investigation.

## Conclusions

From the perspective of activating pyroptosis, both viruses and bacteria can utilize their effector proteins or PAMPs to activate the inflammasome or employ pathogen-encoded proteases to directly cleave GSDMs, thereby triggering canonical pyroptosis (e.g., SARS-CoV-2 NSP6 and Group A streptococcal M protein trigger NLRP3-dependent pyroptosis, whereas SARS-CoV-2 protease NSP3 and GAS cysteine protease SpeB trigger the activation of pyroptosis by cleaving GSDMs). However, a distinct mechanism is observed in bacteria: *Salmonella* can additionally induce noncanonical pyroptosis through the TLR4 pathway via LPS. In the context of suppressing pyroptosis, both pathogens exhibit overlapping strategies, primarily by inhibiting inflammasome assembly and targeting GSDMs to block pyroptotic cell death (e.g., CMV M84 protein and *M. tuberculosis* PknF inhibits inflammasome formation or activation, hence blocking pyroptosis, SVV 3C circumvents GSDMA-mediated pyroptosis by targeting the pore-formation domain). In contrast, bacteria employ mechanistically distinct strategies to suppress pyroptosis, such as ubiquitination-mediated degradation of GSDMD or disrupting its membrane localization through the host ubiquitin system (e.g., *Shigella* IpaH7.8 ubiquitinates GSDMD for proteasomal degradation, *M. tuberculosis* PtpB binds to host ubiquitin to dephosphoryze host plasma membrane phosphoinositides, thereby preventing pyroptosis by limiting the membrane localization of GSDMD-N).

A complicated interaction between the infection and the host characterizes the immune response to intracellular pathogens. While ambient bacteria are successfully destroyed by the host’s defense mechanisms, pathogens that have adapted to their host can avoid the host’s immune system. In this immune response, pyroptosis plays a double-edged sword, which, on the one hand, releases danger signals that activate the subsequent inflammatory response, thereby eliminating the replicative niches of these pathogens. Conversely, the release of excessive inflammatory factors can result in the development of severe inflammatory diseases in the host. Hence, pathogens have evolved a number of strategies to regulate cellular pyroptosis, enabling them to evade the host immune system while also facilitating replication and sustained infection. And a substantial body of evidence from in vivo studies indicates that drugs targeting cellular pyroptosis can effectively inhibit pathogen replication and attenuate the inflammatory response [112, 114, 118]. The recent discovery of a new mechanism of pyroptosis mediated by NINJ1 and palmitoylated-GSDMD suggests that future studies may reveal new mechanisms of host–pathogen interactions during pyroptosis. Also, to fully comprehend these immune responses, additional research is necessary to clarify the complex molecular pathways involved. Moreover, when considering pathogen infections, studying the communication between controlled cell death pathways could offer a valuable understanding of the immune response to these pathogens.
